# Efficient differentiation and polarization of primary cultured neurons on poly(lactic acid) scaffolds with microgrooved structures

**DOI:** 10.1038/s41598-020-63537-z

**Published:** 2020-04-21

**Authors:** Asako Otomo, Mahoko Takahashi Ueda, Toshinori Fujie, Arihiro Hasebe, Yoshitaka Suematsu, Yosuke Okamura, Shinji Takeoka, Shinji Hadano, So Nakagawa

**Affiliations:** 1 Micro/Nano Technology Center, Tokai University, Hiratsuka, Kanagawa 259-1292 Japan; 2 Department of Molecular Life Sciences, Tokai University School of Medicine, Isehara, Kanagawa 259-1193 Japan; 3 School of Life Science and Technology, Tokyo Institute of Technology, B-50, 4259 Nagatsuta-cho, Midori-ku, Yokohama, Kanagawa 226-850 Japan; 4 PRESTO, Japan Science and Technology Agency, 4-1-8 Honcho, Kawaguchi-shi, Saitama, 332-0012 Japan; 5 Graduate School of Advanced Science and Engineering, Waseda University, TWIns, 2-2, Sinjuku-ku, Tokyo, 162-8480 Japan; 6 Department of Applied Chemistry, School of Engineering, Tokai University, Hiratsuka, Kanagawa 259-1292 Japan; 7 Waseda Research Institute for Science and Engineering, Waseda University, Shinjuku-ku, Tokyo 169-8555 Japan

**Keywords:** Bioinformatics, Cell culture, Biomaterials - cells, Cell polarity

## Abstract

Synthetic biodegradable polymers including poly(lactic acid) (PLA) are attractive cell culture substrates because their surfaces can be micropatterned to support cell adhesion. The cell adhesion properties of a scaffold mainly depend on its surface chemical and structural features; however, it remains unclear how these characteristics affect the growth and differentiation of cultured cells or their gene expression. In this study, we fabricated two differently structured PLA nanosheets: flat and microgrooved. We assessed the growth and differentiation of mouse primary cultured cortical neurons on these two types of nanosheets after pre-coating with poly-D-lysine and vitronectin. Interestingly, prominent neurite bundles were formed along the grooves on the microgrooved nanosheets, whereas thin and randomly extended neurites were only observed on the flat nanosheets. Comparative RNA sequencing analyses revealed that the expression of genes related to postsynaptic density, dendritic shafts, and asymmetric synapses was significantly and consistently up-regulated in cells cultured on the microgrooved nanosheets when compared with those cultured on the flat nanosheets. These results indicate that microgrooved PLA nanosheets can provide a powerful means of establishing a culture system for the efficient and reproducible differentiation of neurons, which will facilitate future investigations of the molecular mechanisms underlying the pathogenesis of neurological disorders.

## Introduction

Dissociated primary neuronal cultures are widely used not only for basic neuroscience research but also for drug discovery for neurological disorders^1–3^. In such culture systems, scaffolds are one of the key factors providing the cells with structural support for attachment and subsequent growth and differentiation. Thus far, numerous synthetic polymers including polystyrene, poly(lactic acid) (PLA), poly(glycolic acid), and poly(lactic-co-glycolic acid)^4–6^ have been developed to serve as scaffolds. Among these, PLA, a biodegradable and resorbable polyester, has recently come into the limelight for its utility in medical applications such as tissue regeneration^7^.

Polymeric ultrathin film consisting of PLA, hereinafter called “PLA nanosheet,” is a thin, soft, and flexible material, with properties that allow it to adhere anywhere without any adhesive materials^8^. Many studies have demonstrated that nanosheets can be used to dress wounds to avoid suture, prevent infection, promote bone regeneration, etc. for biomedical applications^8–13^. Nanosheets are also suitable for use as a sheet substrate in cell culture for several reasons. First, nanosheets can easily adhere to the surface of standard culture plates, culture dishes, and cover glass without any adhesive materials. Second, cells and/or tissues cultured on nanosheets can be easily recovered, allowing researchers to easily analyze biological molecules such as proteins, DNA, and RNA. Last, a variety of structural patterns of the nanosheet surface, such as grooves and pores, is possible^14,15^.

Despite these advantages, there are a number of issues that can hinder the application of nanosheets to cell culture experiments. The surface of the nanosheet is hydrophobic, which prevents cell adhesion. Therefore, surface pre-treatment of the nanosheets is required^16–18^. For cells, particularly dissociated neurons, cell adhesion molecules such as poly-D-lysine (PDL) peptides, which confer a positive charge on the nanosheet surface and assist cell adhesion^19^, are required. Further, dissociated cultured neurons under standard culture conditions^20^ extend neurites in random directions, which prevents neurons from forming organized neuronal networks. This may mainly be due to a lack of appropriate attractive and repulsive biological cues from the surrounding cells as well as an absence of scaffold-linked mechanical cues to guide the direction of axon pathfinding. Moreover, it has been shown that morphogenesis in cultured neurons can be affected by topographical differences on the PLA substrate, and grooved structures, in particular, may improve the guidance of neurite extension^21^, although the molecular mechanisms underlying such phenomena remain to be investigated.

In this study, we fabricated microgrooved nanosheets with different microgroove widths and used flat nanosheets as the control. After coating with cell adhesion molecules, we then investigated the effects of the topographical features of these nanosheets on the morphology of mouse primary cultured cortical neurons. Further, to elucidate the molecular basis of the observed differences, we compared the gene expression profiles in cell cultures on two types of nanosheets. Our findings indicated that microgrooved nanosheets served as an effective scaffold for the controlled neurite polarization of cultured neurons, thereby promoting the efficient and reproducible differentiation of neurons. Thus, microgrooved nanosheets are expected to be applied to a large number of investigations in neuroscience research as well as regenerative medicine.

## Results and Discussion

### Assessment of materials used to pre-coat the nanosheet surface

The surface of a PLA nanosheet is smooth and hydrophobic, thereby preventing cell adhesion^16,18^. To establish the appropriate conditions for pre-coating the surface of the nanosheets, we assessed PDL and PDL coated over truncated recombinant human vitronectin (PDL + VTN-N). Vitronectin (VTN-N) is a recombinant human protein that can provide a defined surface for feeder-free culture of human pluripotent stem cells^22^. VTN-N is an extracellular matrix molecule that supports neurite outgrowth *in vitro* both under normal conditions and after trauma^23^. We then cultured PC12 cells, a cell line derived from pheochromocytoma in the rat adrenal medulla, on two different substrates: glass coverslips or nanosheets. Both substrates were pre-coated with PDL or PDL + VTN-N, since neuronal cells hardly adhered to those substrates without cell adhesion molecules.

**Figure 1:**
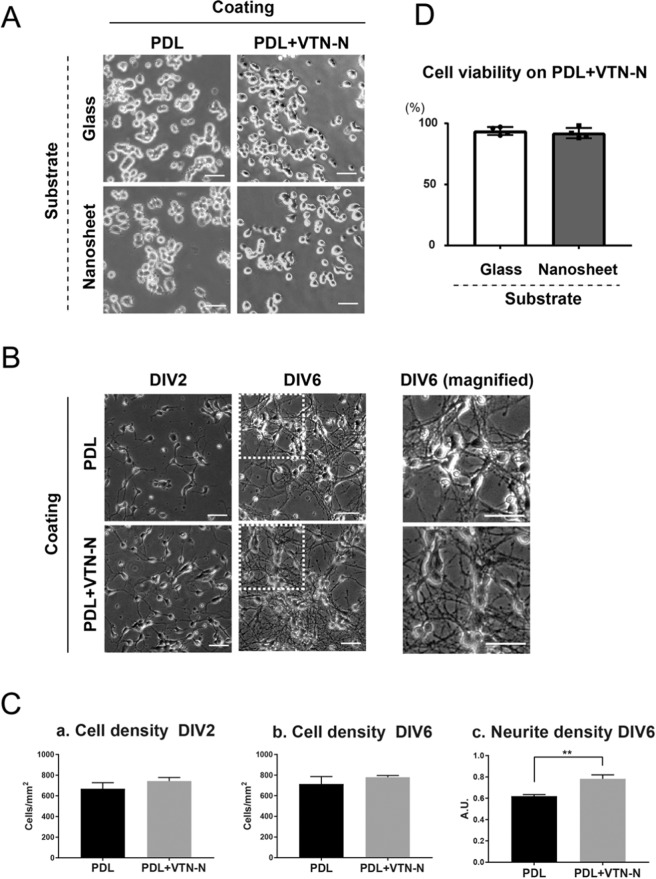
Culturing neurons on the PLA nanosheet. (**A**) PC12 cells cultured on a glass substrate or PLA nanosheet. The glass substrate and PLA nanosheet were coated with PDL or PDL + VTN-N. Scale bars, 50 µm. (**B**) Mouse primary cultured cortical neurons on PDL- or PDL + VTN-N-coated PLA nanosheet. Neural morphology at DIV2 and DIV6 are shown. Mouse primary cultured cortical neurons displayed more prominently elongated neurites on the PDL + VTN-N-coated PLA nanosheet at DIV6 (see enlarged images). Scale bars, 50 µm. (**C**) Differences in coating materials did not affect the density of primary cultured cortical neurons at DIV2 [PDL: 668.1 ± 59.49, PDL + VTN-N: 743.7 ± 33.93 cells/mm^2^ (mean ± SE)] and DIV6 [PDL: 713.9 ± 72.1, PDL + VTN-N: 780.6 ± 16.43 cells/mm^2^ (mean ± SE)], whereas the neurite density at DIV6 was significantly increased by PDL + VTN-N coating (**p < 0.01 unpaired t-test). (**D**) Comparison of the viabilities of mouse primary cultured neurons between on the glass substrate and the PLA nanosheet. The viability of neurons on the PLA nanosheet was comparable with that on the glass substrate [glass: 93.75 ± 1.65, nanosheet: 92.00 ± 2.16 (mean ± SE), expressed as a percentage of the cell viability on plastic culture dishes coated with PDL + VTN-N].

PC12 cells attached and grew on the glass as well as the nanosheets that were pre-coated with either PDL or PDL + VTN-N (Fig. 1A). We also confirmed that mouse primary cultured cortical neurons grew and differentiated on the nanosheets coated with PDL or PDL + VTN-N as observed for the PC12 cells (Fig. 1B). At 2 days *in vitro* (DIV2), cell adhesion and neurite protrusions were observed for mouse primary cortical neurons on the nanosheet coated with either PDL or PDL + VTN-N (Fig. 1B), like those on the glass substrate (Fig. S1). The difference in coating molecules did not affect the cell density at DIV2 or 6 days *in vitro* (DIV6) (Fig. 1C-a-b). However, at DIV6, neuron-extended neurites began to connect to each other (Fig. 1B), suggesting that normal differentiation of neurons was achieved on the nanosheet. Interestingly, at this time point, PDL + VTN-N coating appeared to more efficiently promote neurite outgrowth and branching than PDL coating (magnified images in Fig. 1B). Indeed, quantitative analyses revealed that PDL + VTN-N coating significantly enhances neurite density on the cell culture surface (Fig. 1C-c), suggesting a promotive effect of VTN-N on neurite outgrowth and branching. Finally, we tested whether the cell viability of the cultured cortical neurons on the PLA nanosheet with PDL + VTN-N was comparable with that on the glass substrate with PDL + VTN-N (Fig. 1D) indicating, no obvious toxicity of the nanosheet in primary cultured cortical neurons. Taken together, the nanosheet coated with PDL + VTN-N is likely suitable for neuronal cultures.

### Development of the microgrooved nanosheet

To develop the nanosheet with a microgrooved surface that was applicable to neuronal cultures, we first sought to fabricate nanosheets with three different surface structures, of which the parallel microgrooves were 20, 30, or 50 μm wide with a height of 6 μm, according to previously reported procedures^24–26^ (Fig. S2). In brief, PLA nanosheets were prepared by spin coating of the PLA-dichloromethane solution on a polydimethylsiloxane (PDMS) negative replica with microgrooved motifs. The micropatterned PLA nanosheets were overlaid with a poly(vinyl alcohol) (PVA) supporting layer and then released from the PDMS mold. The nanosheets with the PVA supporting layer were immersed into phosphate-buffered saline without Mg^2+^ or Ca^2^ + [PBS( − )] and then captured by a glass substrate.

Next, to assess the quality of the processed nanosheets, we measured the fine structure of each surface with a Dektak stylus profiler (Bruker, Billerica, MA, USA; Fig. S3). It was revealed that nanosheets with 50 μm wide parallel microgrooves were most stably and reproducibly fabricated, whereas those with 20 or 30 μm in width were not (Fig. S3). Therefore, we decided to use nanosheets with 50 μm wide parallel microgrooves for subsequent experiments.

### Morphological analysis of neuronal cells cultured on microgrooved nanosheets

**Figure 2:**
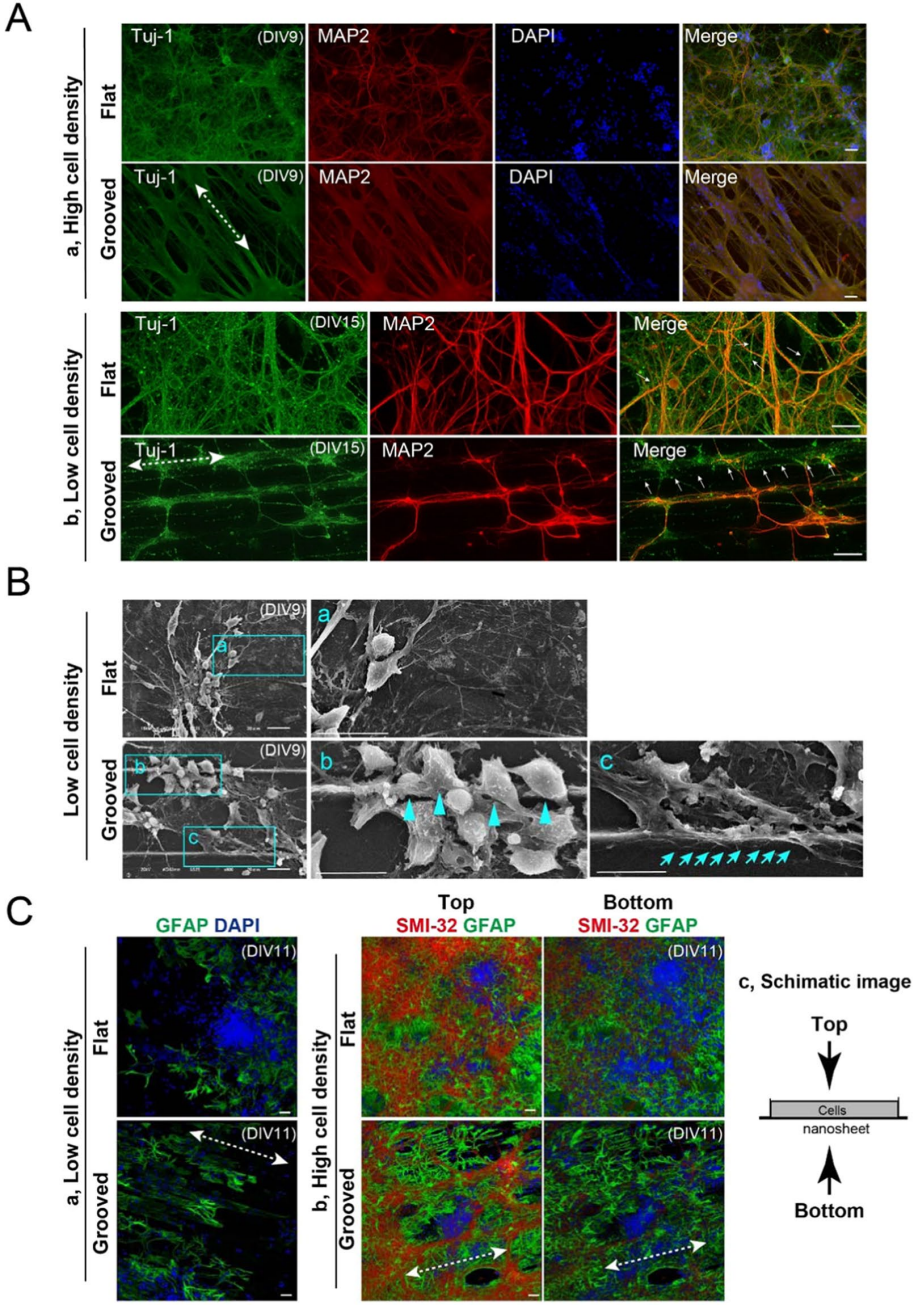
Culturing neurons on the flat and microgrooved PLA nanosheets. (**A**) Morphological analysis of neural cell culture on nanosheets. Tuj-1 and MAP2 were used as neurite and dendrite markers, respectively. Nuclei were visualized by DAPI staining. A dashed line with double-headed arrows indicate the direction of microgroove processing. Under high cell density conditions at DIV9, cultured neurons on the flat nanosheet extended neurites in random directions (a, top-panels) at DIV9, while those on the microgrooved nanosheet extended neurites along the microgrooves (a, bottom-panels). Under low cell density conditions at DIV15, MAP2-negative/Tuj-1-positive axons were detected both on the flat and microgrooved PLA nanosheets (b, indicated as arrows). Interestingly, axons were elongated along the microgroove processing, although cell bodies adhered to the sidewall of microgroove structures on the microgrooved PLA nanosheet (b, bottom-panels). Scale bars, 50 μm. (**B**) SEM analysis of mouse primary cultured cortical neurons on flat and microgrooved PLA nanosheets at DIV9. Neurons on the flat nanosheet adhered to each other and extended neurites from the cell body (a). Neurons on the microgrooved nanosheet densely colonized the inside of the microgrooves between the vertical ridges. Interestingly, neurons also adhered to the sidewall of the ridges rather than to the bottom of the microgrooves (b, indicated by blue arrowheads) and then extended neurites (c, indicated by blue arrows). Scale bars, 20 μm. (**C**) Localization of astrocytes and neurons on the flat and microgrooved PLA nanosheets at DIV11. Under low cell density conditions, GFAP-positive astrocytes adhered to the bottom of the grooves and aligned along the microgrooves on the microgrooved nanosheet. The orientation of microgrooves was indicated by a dashed line with double-headed arrow (a). Under high cell density conditions, astrocytes adhered to bottom side on the nanosheet, while SMI-32-positive neurons seemed to attach onto the top side of astrocytes on both flat and grooved nanosheets. See Supplementary Information Movie 1 and 2 (b). Schematic image of the orientation of cell culture was shown (c).

To assess the effects of the surface microstructure of the nanosheet on the cell adhesion, neurite outgrowth, and morphology of the cultured neurons, we cultured mouse primary cortical neurons on nanosheets with either a flat or parallel-microgrooved surfaces that were pre-coated with PDL + VTN-N. In this experiment, we cultured cortical neurons at a high or low cell density (9 × 10^5^ cells/cm^2^ and 2 × 10^5^ cells/cm^2^, respectively) to monitor the morphology of neurons under both conditions. At 9 and15 DIV, we fixed and stained the cells with anti-MAP2 and anti-Tuj-1 antibodies and investigated their neurite orientations and morphologies on the nanosheet. Tuj-1 and MAP2 are neurite and dendrite markers, respectively. Cultured neurons on the flat and microgrooved nanosheets were both positive for MAP2 and Tuj-1, suggesting that the cells firmly attached to and fully differentiated on the nanosheets irrespective of their surface structure (Fig. 2A). Notably, under the high cell density conditions (Fig. 2A-a), cultured neurons on the microgrooved nanosheet extended neurites along the direction of the parallel microgrooves (indicated by a dashed line with double-headed arrow) and formed thick neurite bundles. Although mechanism of neurite bundle formation on the microgrooved nanosheet is unclear, it is possibly that the axonal contact to substrate topology, like *in vivo* axonal guidance affects those phenotypes^27^. By contrast, neurons on the flat nanosheet extended thin and separated neurites in random directions (Fig. 2A-a). Under the low cell density conditions, we detected Tuj1-positive/MAP2-negative axons on both flat and microgrooved nanosheets (Fig. 2A-b, indicated by white arrows). Interestingly, those axons elongated along microgroove structures on the nanosheet (Fig. 2A-b, indicated by white arrows). By contrast, MAP2-positive cell bodies adhered to the sidewall of the ridges in addition to the bottoms of the microgrooves (Fig. 2A-b). These localization patterns were uniformly observed throughout the microgrooved nanosheet (Fig. S4).

To examine how the grooved structure serves as a structural scaffold for neurons in detail, we investigated the locations where neurons adhered to the microgroove structures at a low cell density by using scanning electron microscopy (SEM) (Fig. 2B). Neurons on the flat nanosheet adhered to each other and elongated neurites from the cell body in random directions (Fig. 2B-a). By contrast, neurons on the microgrooved nanosheet densely colonized the inside of the microgrooves between the vertical ridges (Fig. 2B). Consistent with immunocytochemistry results, enlarged images revealed that some neurons adhered to the sidewall of the ridges (Fig. 2B-b, indicated by arrowheads), and then extended neurites (Fig. 2B-c, indicated by arrows). These results suggest that neurons may also use the vertical surface of the ridges as scaffolds in addition to the bottom surface of microgrooves. Thus, prior to the microgrooves on a nanosheet affect the architecture of cell-to-substrate adhesion, thereby changing the neurite morphology and, presumably, also its biological function.

Because astrocytes also act as scaffolds for neurons *in vivo* and support cell adhesion, neurite outgrowth, and maturation, we investigated the localization of astrocytes on the microgrooved nanosheet. We first analyzed them under low cell density conditions (Fig. 2C-a). On the flat nanosheet, GFAP-positive astrocytes adhered to the surface of nanosheet (Fig. 2C-a). On the microgrooved nanosheet, astrocytes adhered to the bottom surface of the microgroove structures, and they were detected along the microgrooves (Fig. 2C-a, indicated by a dashed line with double-headed arrow). Unlike neurons, astrocytes were localized in the bottoms of the microgrooves rather than to the sidewall. At a high cell density, astrocytes adhered to the surface of the nanosheet on both the microgrooved and flat nanosheets. Further, the localization pattern of astrocytes was not affected by the microgroove structures (Fig. 2C-b). Notably, the major neurons adhered to astrocytes rather than to the substrate (Fig. 2C-b and -c, Supporting information Movie 1 and Movie 2), suggesting that astrocytes supported neurons to adhere on both flat and microgrooved nanosheet. The observation from the top of cell culture (Fig. 2C-c) demonstrated that SMI-32, a marker for neuron, extended along the microgroove structures (Fig. 2C-b, indicated by a dashed line with double-headed arrow, see Fig. S5). Consistently, our high magnification images confirmed SMI-32 positive neurite extended along the microgroove structures (Fig. S6A). In addition, quantification of angle measurement of the SMI-32 positive neurite indicated that the direction of microgroove structures controlled neurite orientation (Fig. S6B). These results suggest that microgroove structures, including the side walls of the microgroove ridges, serve as scaffolds for neurons in addition to astrocytes. Our observation also implies that location of neuron adherence influences the direction of neurite outgrowth and elongation along the microgrooved structures.

### Gene expression patterns of neuronal cells cultured on the nanosheets

**Figure 3:**
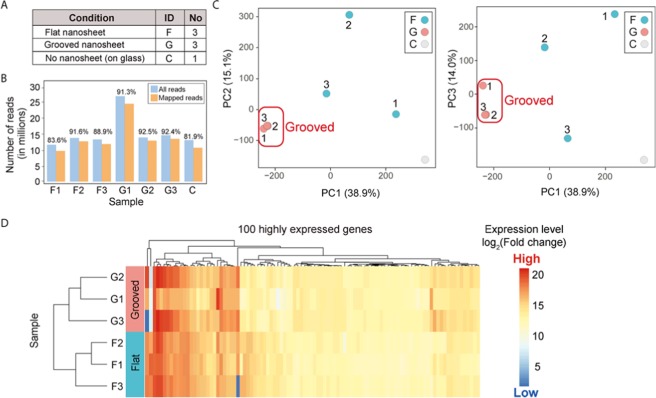
RNA-Seq analysis. (**A**) Summary of the samples analyzed in the RNA sequencing (RNA-Seq) study. (**B**) The number of reads per sample generated by RNA-Seq is shown. Sample names on the x-axis are the same as those in (**A**). A color bar indicates the number of all (blue) or mapped (orange) reads for each sample. The value above each bar represents the percentage of mapped reads. (**C**) PCA plots of PC1, PC2, and PC3. The percentile contribution of each component’s variance is shown on each axis. The numbers above each data point indicate the numbers of the sample name. (**D**) Heatmap of the normalized expression values of the top 100 highly expressed genes in the six nanosheet-cultured samples. The color indicator represents the regularized log-transformed gene expression data.

To understand the molecular basis of the observed morphological differences in neuronal cells between the flat and microgrooved nanosheets, we performed whole exome sequencing (RNA-Seq) analyses on the primary cultured mouse cortical neurons. Seven different samples were subjected to RNA-Seq analysis: flat nanosheet, n = 3; microgrooved nanosheet, n = 3; and glass coverslip, n = 1 (Fig. 3A). For each sample, 75-bp paired-end sequencing reads were mapped to the mouse reference genome (mm10), and the results are summarized in Fig. 3B. To examine gene expression levels among different samples, the number of mapped reads was normalized by regularized log transformation implemented in DESeq2 program^28^. Using the normalized expression values, we assessed the overall similarity of gene expression among the seven samples by principal component analysis (PCA). PCA is a dimension-reduction method for transforming a large set of variables to small sets of principal components (PCs), which represent all the variables of a given dataset. Thus, in a PCA plot, samples forming a cluster indicate that their variations are explained by the PCs with similar patterns. PCA of the RNA-Seq data showed that the cells on the microgrooved nanosheet formed a distinct data cluster, whereas the other samples did not (Fig. 3C). This indicated that neurons on the microgrooved nanosheet exhibited a consistent pattern of gene expression. The proportion of variance, which indicates how much variance was explained by each component, was 38.9%, 15.1%, and 14.0% in PC1, PC2, and PC3, respectively (Fig. 3C), demonstrating that these three PCs captured approximately 68% of the gene expression variance in the samples. Further hierarchical cluster analysis confirmed that the expression pattern of neurons on the microgrooved nanosheet was closely clustered compared with other samples (Fig. S7). We calculated the Euclidean distance among samples based on the normalized expression level of each gene and clustered them using the furthest neighbor method. As expected, 3 samples on the grooved nanosheet were clustered first, followed by the other samples. The distinct differences in the gene expression pattern between nanosheet groups was also observed when using normalized gene expression values of the top 100 highly expressed genes (Fig. 3D). The results suggested that the gene expression patterns of cells on microgrooved were clearly distinct from those on the flat nanosheets. These results combined with the morphological findings support the notion that microgrooves on the nanosheet affect the gene expression of neuronal cells and facilitate their stable differentiation.

### Differentially expressed gene analysis in neuronal cells cultured on nanosheets

**Figure 4:**
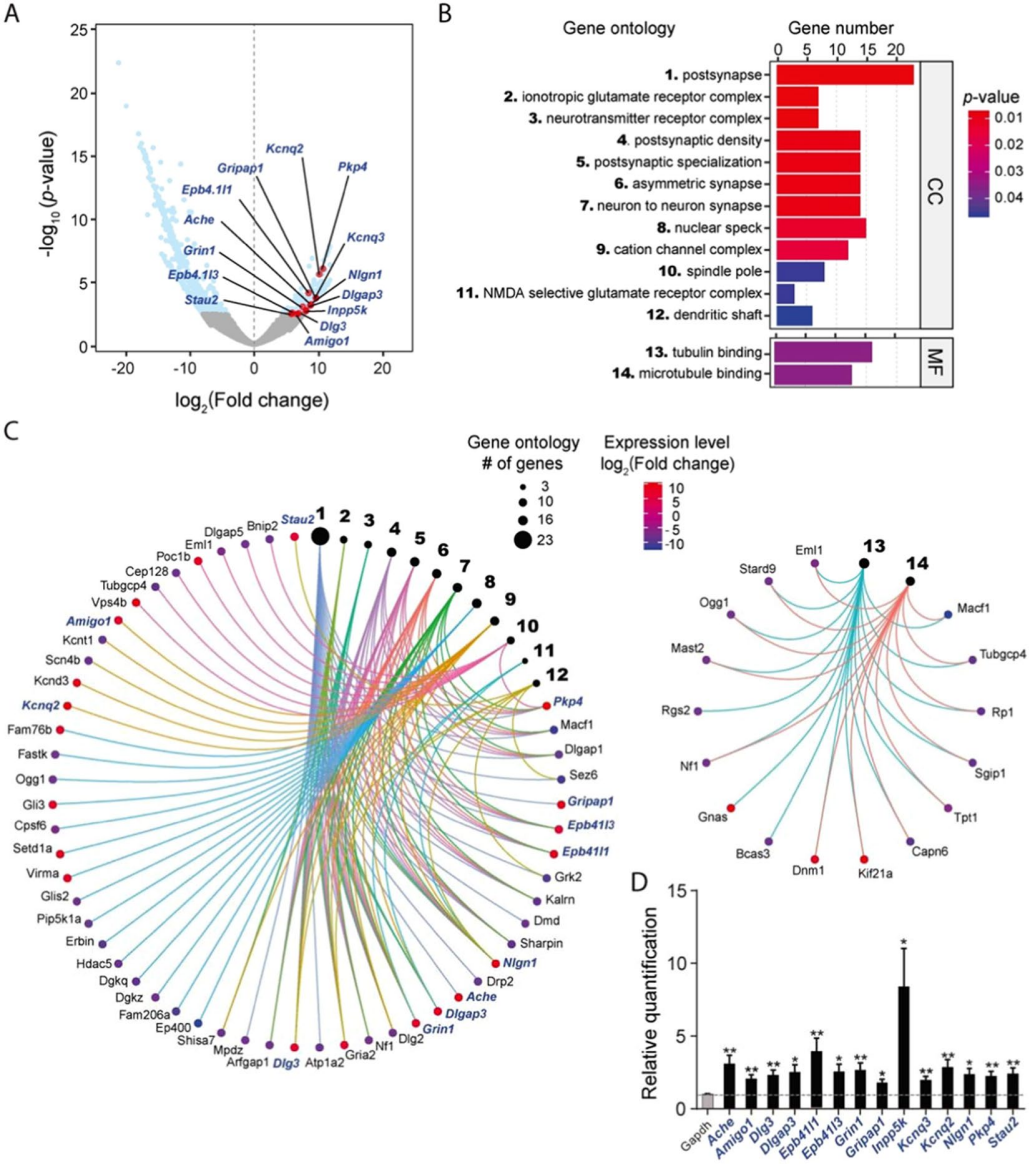
qPCR analysis of the DEGs. (**A**) Volcano plot of the *p* value as a function of the log2-transformed fold changes for each gene in the samples cultured on the microgrooved versus flat nanosheets. The light blue dots represent statistically up or down-regulated transcripts identified by DEG analysis [*p*  <  0.05, Benjamini–Hochberg (BH) corrected]. The genes validated by qPCR are highlighted in red. (**B**) Enriched gene functions of DEG identified by Gene Ontology analysis with adjusted *p* < 0.05 (BH corrected): CC, cellular component; MF, molecular function. (**C**) Gene-Concept Network of the CC (left) and MF (right) DEG, showing the relationship between the GO categories and the genes. The black circles represent the GO categories, whose functions are indicated by the same numbers used in (**B**). The size of the circles for each GO category corresponds to the number of genes in the category. The value of the log2-transformed fold change of each gene is indicated by the color bar. Blue italics indicate genes validated by qPCR. (**D**) Relative gene expression levels of selected DEGs. Genes *Ache*, *Amigo1*, *Dlg3*, *Dlgap3*, *Epb41l3*, *Epb41l1*, *Grin*, *Gripap1*, *Inpp5k*, *Kcnd3*, *Kcnq2, Nlgn1*, *Pkp4*, and *Stau2* in the samples from the flat and microgrooved nanosheets were quantified by qPCR. The dotted line represents the gene expression of samples on the flat nanosheet. Values are mean ± SE. Statistically significant differences between the flat and microgrooved nanosheets are indicated by asterisks (**p < 0.01, *p < 0.05). Expression of *Gapdh* was used as an internal control.

To identify genes associated with the observed differences in the morphological features of neuronal cells between flat and microgrooved nanosheets, we performed differentially expressed gene (DEG) analysis. A total of 300 increased and 1,458 decreased DEGs were identified (Fig. 4A). To investigate the gene functions of the DEGs potentially associated with the differences in the gene expression profiles, we performed Gene Ontology (GO) analysis. Ontology describes gene function with respect to three biological aspects: molecular function (MF), cellular component (CC), and biological process (BP)^29^. On the basis of the GO analysis, we uncovered statistically enriched functional categories of CC and BP (Fig. 4B). In particular, the functions of the up-regulated genes in samples cultured on the microgrooved nanosheet were linked to morphological features of neurons such as the postsynapse, postsynaptic density, dendritic shaft, and asymmetric synapse (Fig. 4B,C). In contrast, the down-regulated genes were mostly linked to tubulin and microtubule binding (Fig. 4B,C).

The up-regulated genes related to the postsynapse, such as *Dlg3*, *Epb41l1*, *Grin1*, and *Nlgn1*, contribute to the formation of the postsynaptic adhesion molecule complex^30^, called the Neuroligin complex. Neuroligins interact with neurexins, a family of presynaptic adhesion molecules, and are major regulators of synapse development and function^31^. Interestingly, *Inpp5k* has been reported to act as a lysophosphatidic acid signaling modulator, and its overexpression promotes the intrinsic axon growth of corticospinal axons^32^. Considering these reports and the results of our NGS analysis, it is presumed that neurons cultured on the microgrooved nanosheet become more mature than neurons cultured on the flat nanosheet.

To validate the up-regulated DEGs identified by RNA-Seq, we performed quantitative RT-PCR (qRT-PCR) on independently prepared mRNA samples (DIV9) from both flat and microgrooved nanosheets. Since NGS analysis revealed that synapse formation and maturation of the neurons were promoted on microgrooved nanosheets, we selected 14 DEGs known to play crucial roles in either postsynapse or presynapse maturation in neurons: *Ache*, *Amigo1*, *Dlg3*, *Dlgap3*, *Epb41l1*, *Epb41l3*, *Grin*, *Gripap1*, *Inpp5k*, *Kcnd3*, *Kcnq2*, *Nlgn1*, *Pkp4*, and *Stau2*. All the selected DEGs were significantly up-regulated in mRNA samples from neuronal cultures on the microgrooved nanosheets compared with those from the flat nanosheets [Ache*, Amigo1**, Dlg3**, Dlgap3*, Epb41l1*, Epb41l3**, Grin**, Gripap1*, Inpp5k*, Kcnd3**, Kcnq2**, Nlgn1*, Pkp4**, and Stau2** (**p < 0.01, *p < 0.05)] (Fig. 4D). These results strongly indicate that microgrooves on a nanosheet can more efficiently facilitate neuronal differentiation, which is consistent with the morphological findings obtained in this study (Fig. 2).

Finally, we analyze whether synapse formation is affected by microgrooves, we conducted immunohistochemical analysis of PSD-95 (postsynaptic density protein 95), a marker for postsynaptic structures. However, quantification of the number of PSD-95 positive puncta in the flat and microgrooved nanosheets reveals no difference between them (Fig. S8A,B, Supplementary Information Movie 3 and Movie 4). The results of RNA-seq supports that microgrooves on a nanosheet can more efficiently facilitate neuronal differentiation. However, after differentiation, microgrooves do not affect the number of matured synapses, as we have shown (Fig. S8A,B, Supplementary Information Movie 3 and Movie 4). Those results imply that although microgrooves on a nanosheet can promote neural differentiation by up-regulating the expression of synapse-related genes prior to the formation of synapses. Cell culture conditions such as local cell density, efficiency of neuronal association with astrocytes, and local concentration of trophic factors from astrocytes might be affected by the presence of microgroove structures, thereby up-regulating the expression of DEGs. In any case, further studies will be required to address this issue.

In this study, we developed a neural cell culture system using a microgrooved PLA nanosheet, which provided a more reproducible and efficient culture environment for the neurons. Thus far, the precise molecular mechanisms by which neuronal maturation is accelerated in the presence of a microgrooved scaffold are still unclear. Nonetheless, this microgrooved nanosheet could provide a powerful means to establish a novel experimental system for neuroscience research and regenerative medicine and may facilitate future investigations of the molecular mechanisms underlying the pathogenesis of many neurological disorders.

## Methods

### Reagents and preparation of nanosheets

All reagents used in this study including the PLA were of analytical grade. Silicon wafers (SiO_2_ substrate; KST World, Fukui, Japan) cut to an appropriate size (typically 3 × 3 cm) were treated with piranha solution, followed by washing with distilled water. PVA (*M*w: 22 kDa; Kanto Chemical, Tokyo, Japan) was dissolved in distilled water at a concentration of 10 mg/mL, and this solution was dropped onto the SiO_2_ substrates and spin-coated at 4,000 rpm for 20 s (Spin Coater MS-A100; Mikasa, Tokyo, Japan), followed by drying at 50 °C for 2 min. A solution of PLA (Mw: 80–100 kDa; Polysciences, Warrington, PA, USA) with the appropriate concentration (ca. 10 mg/mL), adjusted to the targeted thickness (150 nm), was dropped onto the PVA-coated substrates and spin-coated at 4,000 rpm for 20 s, followed by drying at 50 °C for 2 min. The obtained substrates were immersed in distilled water to collect free-standing nanosheets. The nanosheets were scooped up with coverslips and fully dried in a desiccator overnight. PDL or PDL with VTN-N was coated onto the PLA nanosheets immediately prior to use for culture experiments.

### Preparation of microgrooved nanosheets

We prepared PDMS stamps with a microgrooved pattern as previously reported^24–26^. The pattern consisted of microgrooves and ridges with a width of 50 μm and a height of 6 μm. To fabricate a free-standing microgrooved nanosheet, we used the same procedures used for a flat PLA nanosheet. These procedures are summarized in Supplementary Fig. 2.

### Coating of nanosheets

For PDL coating, PDL (molecular weight 70–150 kDa, #P6407; Sigma-Aldrich) at a concentration of 0.1 mg/mL in 0.1 M borate buffer was coated onto the nanosheets at a final surface area coating concentration of 30 μg/cm^2^. The nanosheets were then incubated in a 5% CO_2_ incubator at 37 °C for 2 h. Next, the nanosheets were washed three times with ultra-pure water and dried on a biological clean bench. For PDL and VTN-N coating, dried PDL-coated nanosheets were incubated with 0.1 mg/mL VTN-N diluted in PBS( − ) at 37 °C for 2 h in a 5% CO_2_ incubator. The VTN-N solution was removed prior to sticking the Press-to-Seal Silicone Isolator with Adhesive (Thermo Fisher Scientific) onto the nanosheets to control the density of the cells (see “Cell culture” in METHODS).

### Animals

All animal experimental procedures were approved by The Institutional Animal Care and Use Committee at Tokai University. All animal experiments were conducted in accordance with the Guidelines for the Care and Use of Animals for Scientific Purposes at Tokai University, which was established based on Act on Welfare and Management of Animals in Japan.

### Cell cultures

PC12 cells were cultured in Dulbecco’s Modified Eagle’s medium with High Glucose (Wako) supplemented with 7.5% (w/v) heat-inactivated fetal bovine serum (FBS; PAA Laboratories), 7.5% (w/v) heat-inactivated horse serum (Gibco), 100 U/mL penicillin G, 100 µg/mL streptomycin, and 100 µg/mL sodium pyruvate. Mouse primary cortical neurons were cultured as previously reported^33,34^. In brief, tissues from each embryo were dissected out and immediately placed into 1 mL of ice-cold HBSS( − ). After removing the HBSS( − ) by aspiration, 0.5 mL of 0.25% trypsin-EDTA was added and the embryo was incubated for 15 min at 37 °C. The trypsin-EDTA was removed, and the embryo was washed several times with 20% FBS/neurobasal medium (Invitrogen). Tissue samples were treated with 50 µg/mL DNase I in 20% FBS/neurobasal medium for 10 min at room temperature (RT). After centrifugation at 150 ×*g* for 15 s, the resulting tissue pellets were dissociated in 0.6 mL of 20% FBS/neurobasal medium by pipetting with a flame-sterilized Pasteur pipette. After counting the number of living cells with the trypan blue assay, 9 × 10^5^ cells were placed onto the PDL- and VTN-N-coated nanosheets using the Press-to-Seal Silicone Isolator with Adhesive to control the cell numbers on the nanosheets. The nanosheets were then immersed in neuronal cell culture media [neurobasal medium containing 1× B-27 supplement (Invitrogen), 25 µg/mL insulin (Sigma-Aldrich), 0.5 mM L-glutamine, 50 µg/mL streptomycin, and 50 U/mL penicillin G] and cultured at 37 °C. The medium was then exchanged for fresh medium containing 5% FBS, and the cells were cultured on the nanosheets for another 36 h.

### Cell viability assay

The alamarBlue Cell Viability Reagent (Thermo Fisher Scientific) was added to 10% (v/v) of the medium at DIV6 of the primary cultured neurons. After 6 h incubation, we detected the fluorescence intensity of resorufin with the Spectra Max i3 (Perkin Elmer). Resazurin, a PC of the alamarBlue reagent, is reduced to the highly red fluorescent resorufin in viable cells only. Experiments were repeated four times.

### Quantification of neurite density

To quantify the neurite density, we counted the cell number on images captured from four independent areas on the flat and microgrooved nanosheet by using Image-J cell count plugin. Areas of neurites including cell bodies in the captured images were segmented by using Image-J with segmentation plugin (Treanable Weka Segmentation, https://imagej.net/Trainable_Segmentation). The segmented area corresponding to neurites and cell bodies was measured by using analyze particle tool on Image-J. The sarea was shown as the ratio of coverage. We used four independent images for this analysis.

### Immunocytochemistry

The cells were fixed with 4% (w/v) paraformaldehyde in PBS( − ) pH 7.5 for 30 min at RT and permeabilized with 0.1% (w/v) TritonX-100 in PBS( − ) for 30 min. The primary antibodies used in previous reports^33,34^ and the anti-SMI-32 antibody (Calbiochem) against for non-phosphorylated form of neurofilament (a widely used marker for axons) were diluted in PBS( − ) containing 1.5% (v/v) normal goat serum and incubated with the samples. After washing the cells with PBS( − ), Alexa 594-conjugated goat anti-mouse IgG (1:500; Molecular Probes) or Alexa 594-conjugated goat anti-rabbit IgG (1:500; Molecular Probes) was used for the detection of proteins of interest. Fluorescent signals were captured with the BZ-X fluorescence microscope (Keyence) and processed with Adobe Photoshop (Adobe).

To prepare a 3D reconstructed image, we captured z-stack images on the nanosheets with a thickness of 10 μm by LSM880 (Zeiss) and reconstructed 3D images. Movies showing “Up and Bottom” images of GFAP and SMI-32 staining and PSD-95 staining were prepared by the ZEN black edition (Zeiss).

### Library preparation

Total RNA was extracted from the cultured cells with the RNeasy Plus Micro Kit (Qiagen) according to the manufacturer’s protocol. The quality of the total RNA samples was validated with the RNA 6000 Pico Kit (Agilent) on the Bioanalyzer (Agilent). High-quality RNA samples with an RNA integrity number >9 were used for library preparation. RNA-Seq libraries were prepared with the Encore Complete RNA-Seq DR Multiplex system (NuGEN) in accordance with the manufacturer’s instructions.

### RNA-Seq analysis

Indexed paired-end cDNA sequencing libraries were sequenced by MiSeq (Illumina, San Diego, CA, USA). A total number of 75-bp paired-end reads were sequenced. After trimming the reads with the fastq_quality_trimmer tool in the FASTX-Toolkit (version 0.0.14) using the option (−Q 33, −t 20, −l 30), the reads were then mapped onto the mouse reference genome (mm10) using HISAT2 (version 2.1.0)^35^ with the default options. StringTie (version 1.3.4b)^36^ was used to quantify gene expression. The R package of DESeq. 2 (version 1.18.1)^28^ was used for RNA-Seq differential expression analysis. We first normalized the gene expression values for each sample using regularized log transformation implemented in the DESeq. 2 program. For each gene, the gene expression data of cells cultured on both the flat and microgrooved nanosheets were statistically examined. We assumed that genes differentially expressed on these two types of nanosheets with a statistical significance of *p*  <  0.05 (Benjamini–Hochberg corrected) was indeed a DEG. Enrichment analysis was carried out with the enrichGO function in the R package of clusterProfiler (version 3.7.1)^37^.

### qRT-PCR

qRT-PCR was performed on 10 ng of total RNA using the Thunderbird SYBR qPCR/RT Set (Toyobo) with the specific primers (0.2 µM each) listed in Supporting Information Table S1. The transcript levels were normalized by the amount of *Gapdh* mRNA in each sample.

### Statistical analysis

Statistical analyses were conducted with Prism 7 (GraphPad). Statistical significance was evaluated by ANOVA followed by appropriate post-hoc tests for multiple comparisons between groups.

## Supplementary information

Supplementary information

Supplementary information2

Supplementary information3

Supplementary information4

Supplementary information5

## Data Availability

The RNA-Seq data obtained in this study have been deposited in the DDBJ DRA database (https://www.ddbj.nig.ac.jp/dra/index-e.html) under the accession numbers DRR166653–DRR166659.
