# Supplementary material for: Efficient differentiation and polarization of primary cultured neurons on poly(lactic acid) scaffolds with microgrooved structures
Source: Sci Rep. 2020 Apr 21;10:6716. doi: 10.1038/s41598-020-63537-z (PMC7174324; doi:10.1038/s41598-020-63537-z)
Supplement: Supplementary file 1 — Supplementary information [file 41598_2020_63537_MOESM1_ESM.pdf]

Efficient differentiation and polarization of primary cultured neurons on poly(lactic acid) scaffolds with microgrooved structures

Asako Otomo, Mahoko Takahashi Ueda, Toshinori Fujie, Arihiro Hasebe, Yoshitaka Suematsu, Yosuke Okamura, Shinji Takeoka, Shinji Hadano, So Nakagawa

Supplementary information

Table 1. Primer sets used in this study

Gene name	Accession number	Primer#1	Primer#2
Dlg3	NM_016747.4	5'-GGGACTTCCCTGGGTTAAGTG	5'-GTTTGACTCGGTCCTTCATTGGG
Dlgap3	NM_198618.5	5'-CTCAGATGGTAGCCCCAAGAC	5'-GCTGCTGGTTAAACTCTTCGC
Grin1	NM_008169.3	5'-GATGATGCGCGTCTACAACTG	5'-CCTTCTCTGCCTTGGACTCAC
Nlgn1	NM_138666.4	5'-ATGCTTCCTGTGTGGTTCACT	5'-TCAGCACCGTCATTATCACCT
Amigo1	NM_146137.3	5'-GTGACCCTTCCATACTCCACC	5'-GAGAAGCAGGAACACGGAGAG
Kcnd3	NM_001039347.1	5'-GGCTTTCTTCTCTTCTCCCTTAC	5'-CTTAGGCACCATGTCTCCATATC
Kcnq2	NM_010611	5'-TGGCCATCCAGTCATTTATCC	5'-CCACAACTGAGGGCTCTATTC
Ache	NM_001290010.1	5'-CGTCCTGCCTCAAGAAAGTATC	5'-CCCGTAAACCAGAAAGTAGGAG
Epb41l1	NM_013510.4	5'-CTGCCTCTGTCTGTCTTGTG	5'-CCTCAAGCCTGCTCTAGTTTAG
Epb41l3	NM_013813.2	5'-CGGAAACCCACAGAGTTCATAG	5'-ATGCCTCTCCTCTACCAGTATC
Gripap1	NM_001290455.1	5'-ACCTGGAGAAGCCAACTAAAG	5'-ACCAACACCAGCCACATATAG
Pkp4	NM_026361.2	5'-GCCTCGCTCTGAATACGATAG	5'-CTCTTGCTGGTGAGGAATAGG
Inpp5k	NM_008916.2	5'-CGGTCCACTGCATATAGACTTG	5'-CCAGGATGGTGTGATAGGAATG
Stau2	NM_001111272.1	5'-GACACAATGCTGCGATGAAAG	5'-GCGCAATCTCAAACACTAAGC

Primer sets used for qRT-PCR are listed. The specificity of a primer pair for PCR amplification of the desired sequence was confirmed by the dissociation analysis of amplicons after the real-time PCR experiment.

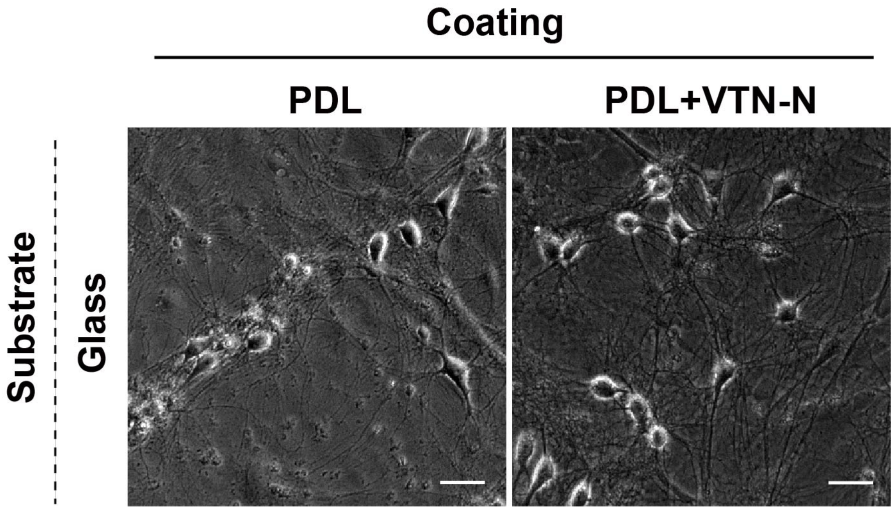

Figure S1. PDL and PDL+VTN-N coating support cell adhesion of primary cortical neurons on a glass substrate. PDL and PDL+VTN-N coating support cell adhesion of primary cortical neurons on the glass substrate.

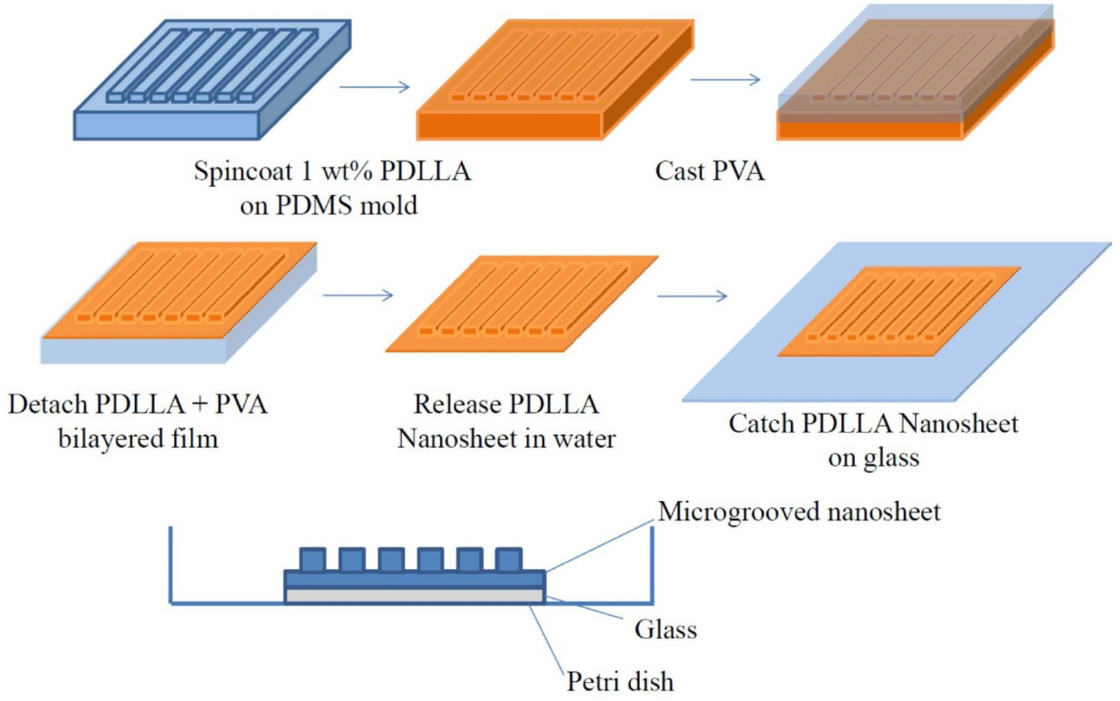

Figure S2. Protocol for the fabrication of the grooved nanosheets

The preparation scheme for the microgrooved PLA nanosheets is shown. PLA nanosheets were prepared by spin coating a 10 mg/mL poly(D, L-lactic acid) (PDLLA) solution onto a PDMS negative replica with grooved motifs. Next, a PVA (MW: 13,000–23,000; Kanto Chemical, Inc., Tokyo, Japan) supporting layer was cast onto the PLA nanosheet. The micropatterned PLA nanosheet with the PVA layer was released from the PDMS mold and then placed into a PBS solution to dissolve the PVA supporting layer.

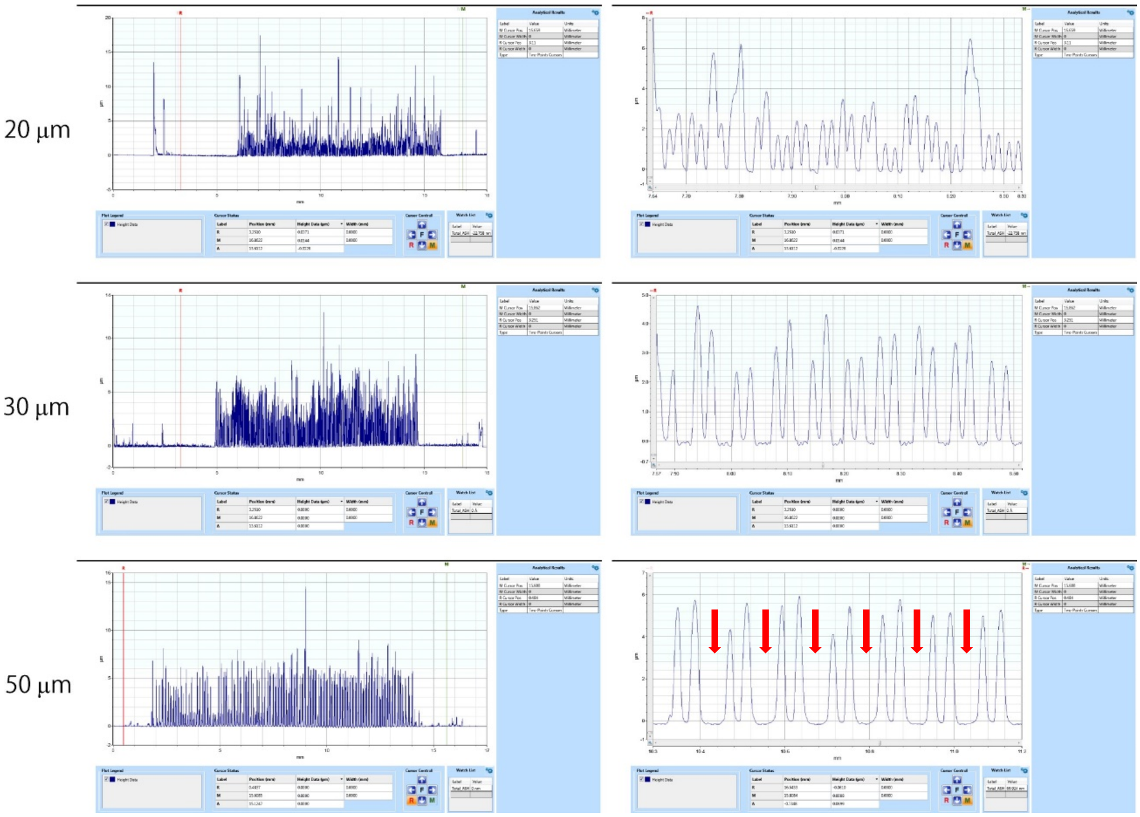

Figure S3. Surface analysis of the grooved nanosheet

The thickness and surface morphology of PLA nanosheets with microgrooves of different widths were analyzed with a surface profiler. Right panels are magnified images of left ones indicated by red squared dotted lines. The X-axis indicates the width of microgroove structures (pointed by red arrows in right panels in showing 50 μm grooves), and Y-axis shows the height of microgroove structures. The microgroove pattern comprising grooves 50 μm in width was the most uniform of the three tested patterns.

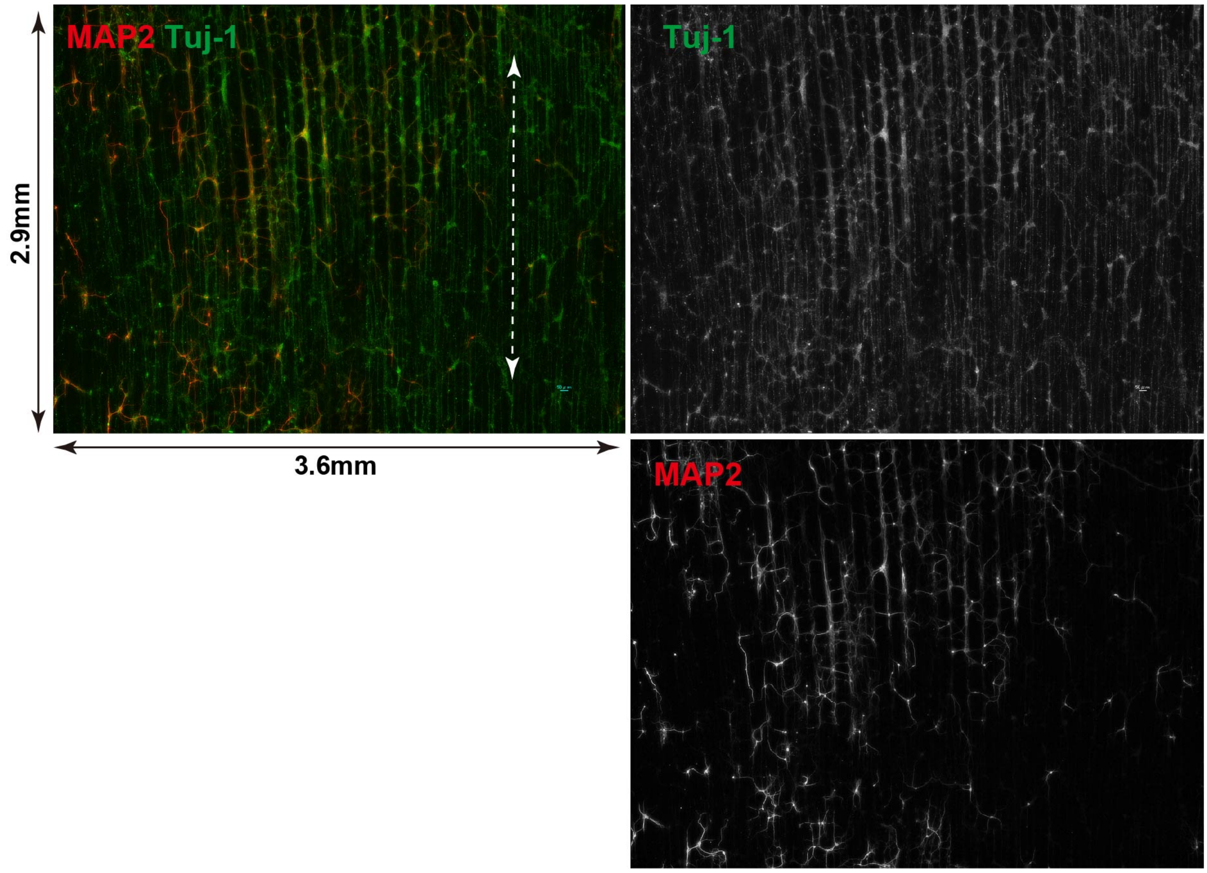

Figure S4. Localization of neurons on PDL+VTN-N coated microgroove nanosheet

To confirm whether neurons adhered to the ridges of the microgrooves, we observed localization of neurons on the microgrooved nanosheet at DIV15. MAP2-positive cell bodies were aligned in the direction of the microgrooves (indicated by a dotted line with double-headed arrow).

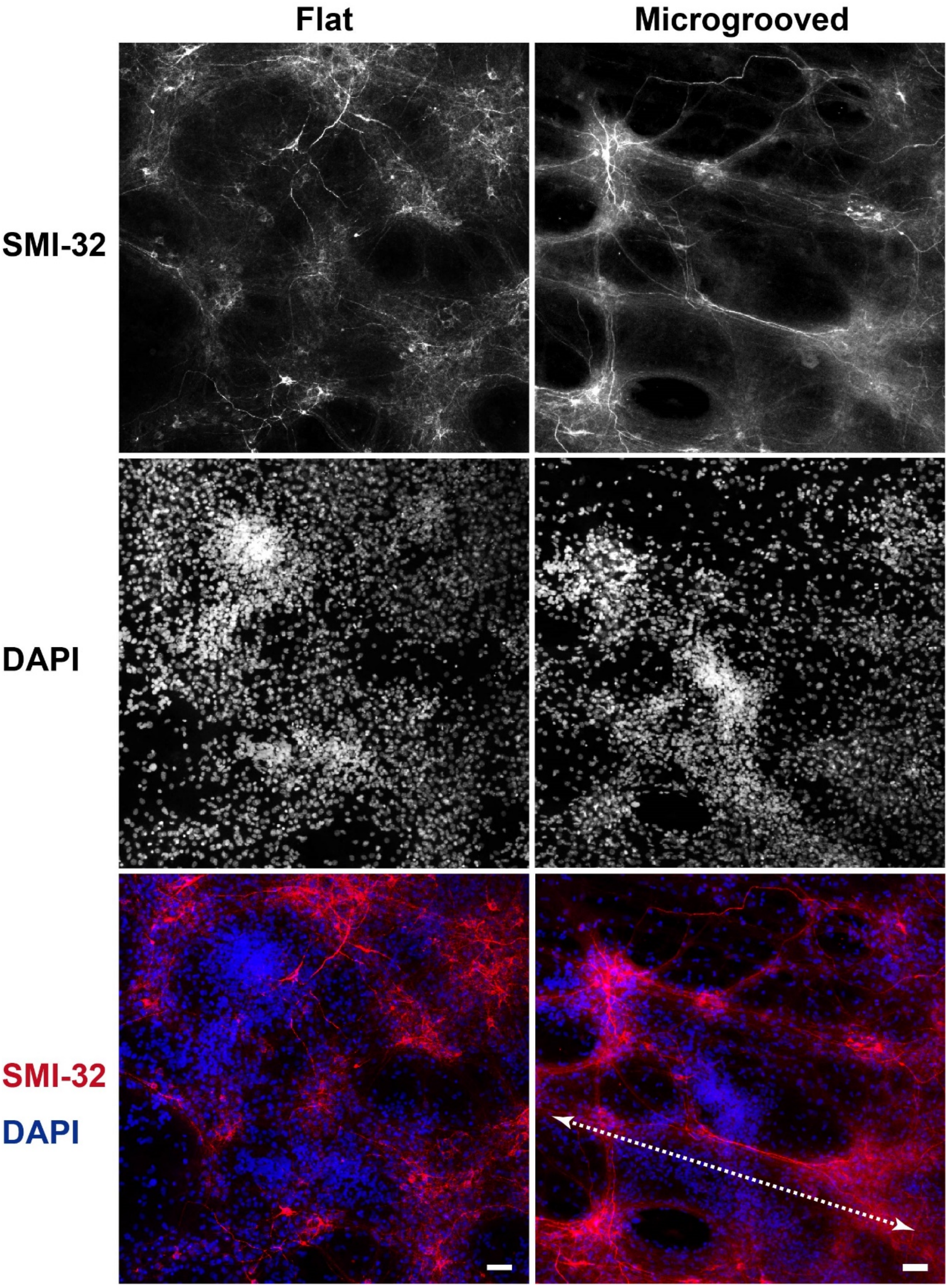

Figure S5. Localization of SMI-32 positive neurons on the microgrooved

nanosheet

SMI-32 immunostaining on the flat and microgrooved nanosheets shown in Figure 2 C-b was enlarged and processed to show the structure of neurons. SMI-positive long neurite corresponding to axon elongated along the microgrooves. The dotted line with double-headed arrows indicates the direction of the microgrooves. Scale bars, 20 μm.

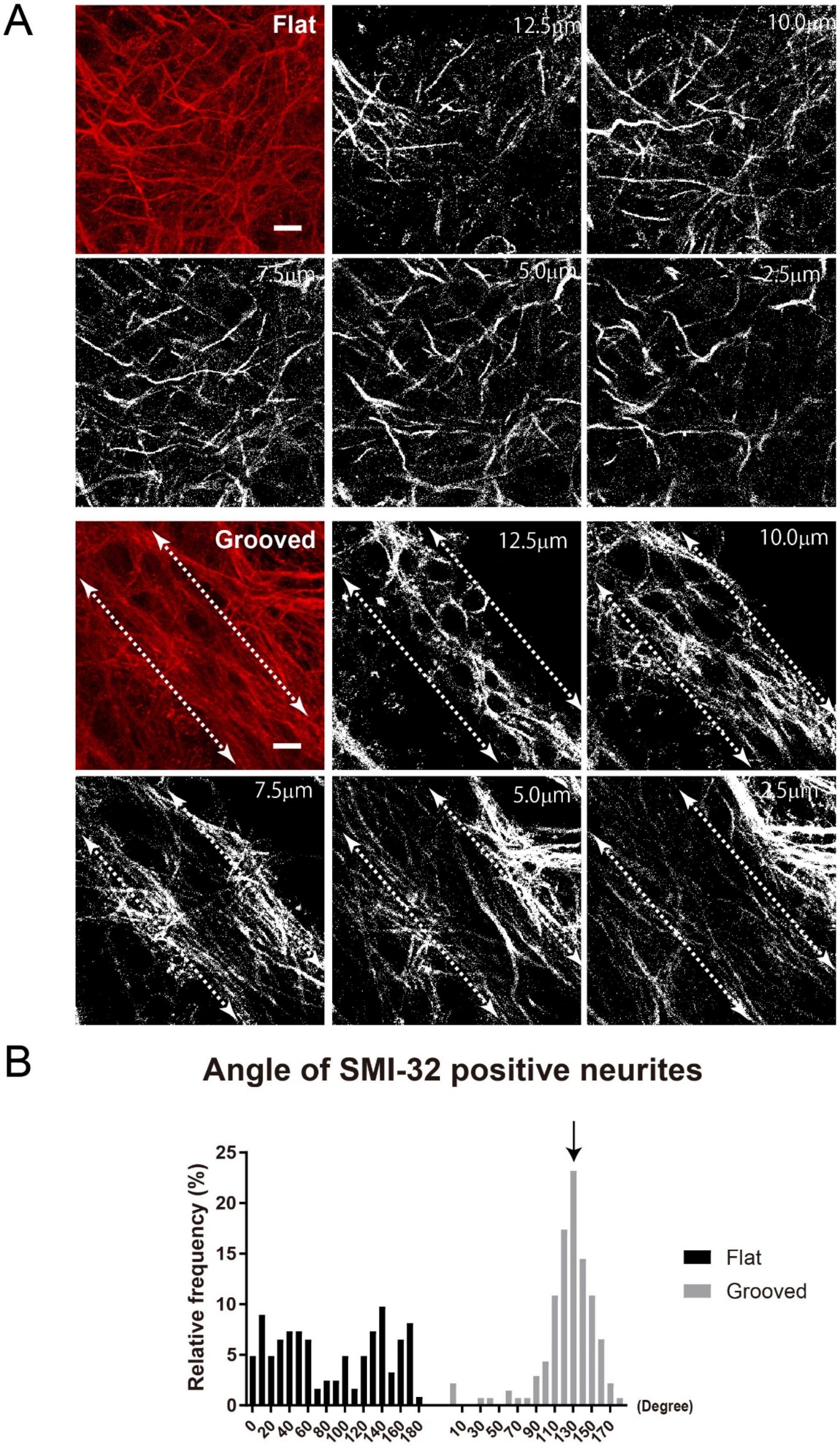
A
Flat
12.5μm
10.0μm
7.5μm
5.0μm
2.5μm
Grooved
12.5μm
10.0μm
7.5μm
5.0μm
2.5μm
B
Angle of SMI-32 positive neurites
Relative frequency (%)
0
5
10
15
20
25
0 20 40 60 80 100 120 140 160 180
10 30 50 70 90 110 130 150 170
(Degree)
Flat
Grooved

Figure S6. Neurite orientation on the microgrooved nanosheet

A) Red fluorescent images show projection images of SMI-32 immunostaining on the nanosheets. Black and white images indicate stack images of SMI-32. 2.5μm thickness images from the bottom (0-2.5μm) to top (10-12.5μm) of cultures are shown. The dotted line with double-headed arrows indicates the direction of the microgrooves angled 130 °on these pictures. On the microgrooved nanosheet, SMI-32 positive neurons adhered to both the bottom surface and sidewall of microgrooves. B) The angle of SMI-32 positive neurites in stack images (shown in Fig.S5A) were measured and tabulated. Neurite longer than 20μm were measured by Image-J. The dotted line with double-headed arrows indicates the direction of the microgrooves angled 130 °on these pictures. The bar graph shows the distribution of neurite angles on the nanosheets. Arrowhead indicates the angle of microgroove structures. Our data demonstrate that the neurite angle converged around 130 ° on the microgrooved nanosheet.

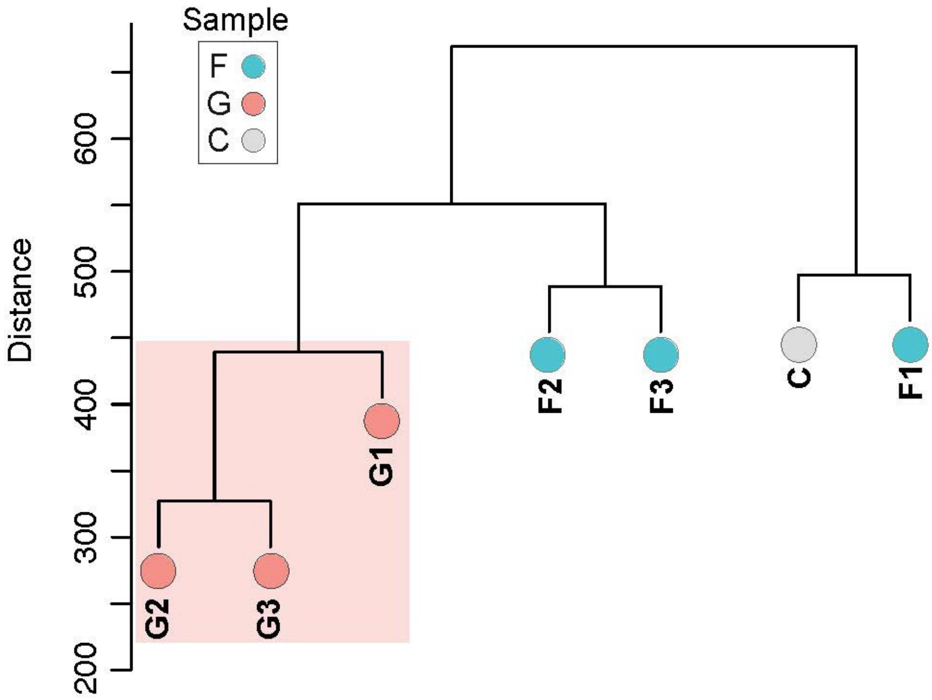

Figure S7. Hierarchical cluster analysis of gene expression profiles

Gene expression profiles shown here correspond to those in Figure 3: F, flat nanosheets; G, grooved nanosheets; C, no nanosheets (on glass).

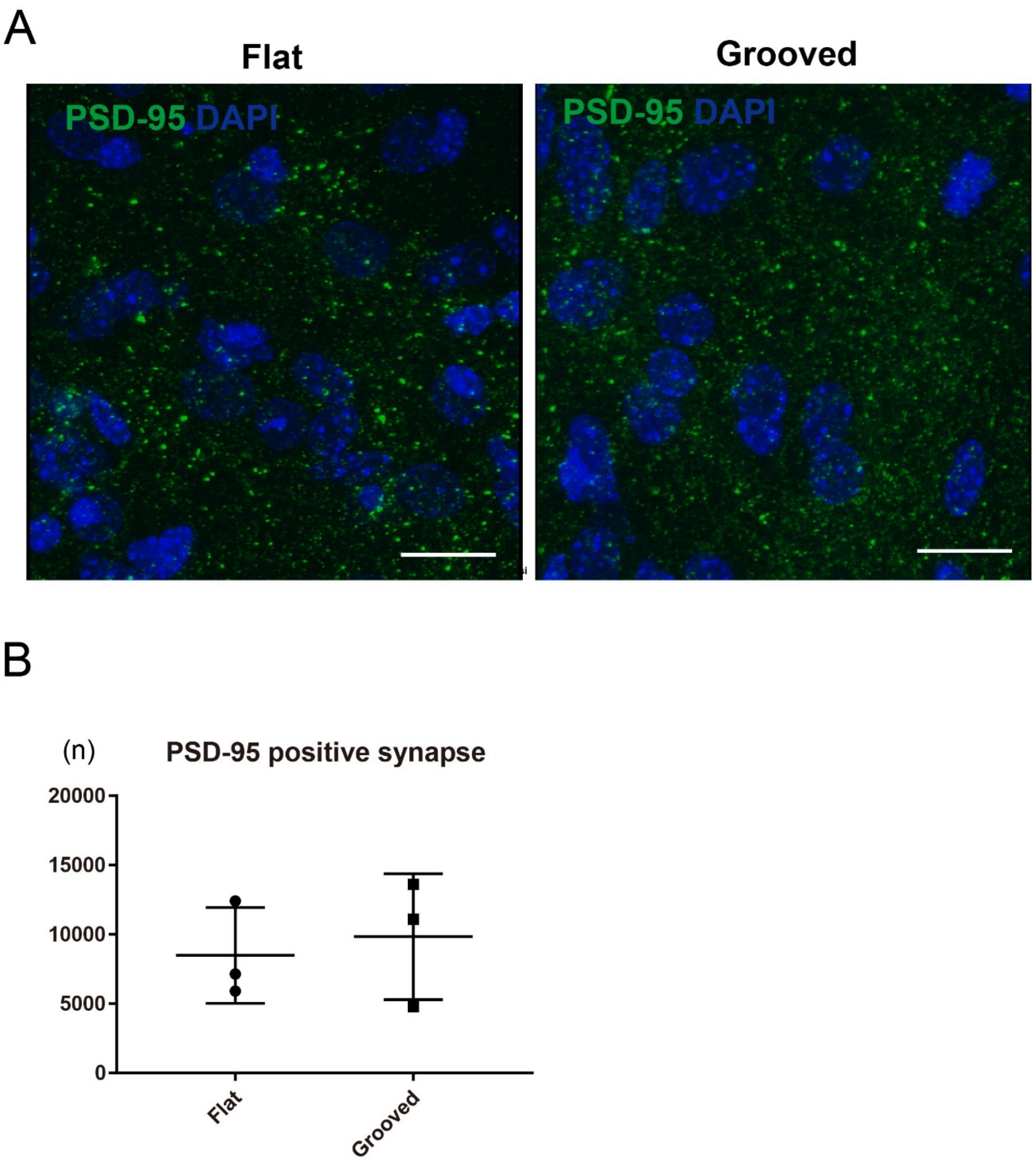

Figure S8. PSD-95 staining in primary cultured cortical neurons on flat and microgrooved PLA nanosheets

A) PSD-95-positive punctuates were detected in the primary cultured cortical neurons

(DIV15) on both flat and microgrooved nanosheets. PSD-95 is accumulated in matured post-synaptic structures. Thus, these images indicate that neurons were fully differentiated and had formed synapses on both flat and microgrooved nanosheets. Scale bar, 20 μm. B) The number of PSD-95-positive post synapses on the flat and microgrooved nanosheets was quantified. Differences in the micropattern of the nanosheets did not affect the average number of post synapses [Flat: 8,486 ± 1,996, Microgrooved: 9,833 ± 2,623 (mean ± SE)] To quantify the number of PSD-positive structures on the culture, z-stack images of PSD-95-positive structures at 10 μm-thickness was captured using LSM700, and 3D images were constructed from the series of images obtained. Quantification of PSD-95-positive structures in a 3D image was prepared by using Image-J 1.52p, analysis "analyze particles."

Movie 1. Distribution of neurons and astrocytes in primary cultured cortical neurons on flat nanosheet.

The movie shows 3D reconstruction data of SMI-32 (red signals) and GFAP (green signals) staining of primary cultured cortical neurons, related to Figure 2.C. Astrocytes adhered to the surface of flat nanosheet (bottom side of cell culture), while neurons appeared to adhere to astrocytes (top side of cell culture).

Movie 2. Distribution of neurons and astrocytes in primary cultured cortical neurons on microgrooved nanosheet.

The movie shows 3D reconstruction data of SMI-32 (red signals) and GFAP (green signals) staining of primary cultured cortical neurons, related to Figure.2C. Astrocytes adhere to the bottom surface of microgrooves (bottom side of cell culture), while neurons were present on the sidewall of microgrooves (top side of cell culture).

Movie 3. PSD-95 staining in primary cultured cortical neurons on flat nanosheets.

The movie shows 3D reconstruction data of PSD-95 (green signals) in primary cultured cortical neurons on flat nanosheet, related to Figure S6A.

Movie 4. PSD-95 staining in primary cultured cortical neurons on microgrooved nanosheets

The movie shows 3D reconstruction data of PSD-95 (green signals) in primary cultured cortical neurons on microgrooved nanosheet, related to Figure S6A.
